# Curcumin-Artemisinin Coamorphous Solid: Xenograft Model Preclinical Study

**DOI:** 10.3390/pharmaceutics10010007

**Published:** 2018-01-09

**Authors:** M. K. Chaitanya Mannava, Kuthuru Suresh, Manish Kumar Bommaka, Durga Bhavani Konga, Ashwini Nangia

**Affiliations:** 1 School of Chemistry, University of Hyderabad, Hyderabad 500 046, India; mchaitanyasharma@gmail.com (M.K.C.M.); kuturusuresh@gmail.com (K.S.); mani071115@gmail.com (M.K.B.); 2 Technology Business Incubator, University of Hyderabad, Hyderabad 500 046, India; 3 Virchow Biotech Pvt. Ltd., Animal House Facility, Hyderabad 500 055, India; kongabilwakshi@gmail.com; 4 Council of Scientific and Industrial Research National Chemical Laboratory, Pune 411 008, India

**Keywords:** curcumin, artemisinin, coamorphous, cocrystal, stability, bioavailability, xenograft

## Abstract

Curcumin is a natural compound present in Indian spice turmeric. It has diverse pharmacological action but low oral solubility and bioavailability continue to limit its use as a drug. With the aim of improving the bioavailability of Curcumin (CUR), we evaluated Curcumin-Pyrogallol (CUR-PYR) cocrystal and Curcumin-Artemisinin (CUR-ART) coamorphous solid. Both of these solid forms exhibited superior dissolution and pharmacokinetic behavior compared to pure CUR, which is practically insoluble in water. CUR-ART coamorphous solid showed two fold higher bioavailability than CUR-PYR cocrystal (at 200 mg/kg oral dose). Moreover, in simulated gastric and intestinal fluids (SGF and SIF), CUR-ART is stable up to 3 and 12 h, respectively. In addition, CUR-PYR and CUR-ART showed no adverse effects in toxicology studies (10 times higher dose at 2000 mg/kg). CUR-ART showed higher therapeutic effect and inhibited approximately 62% of tumor growth at 100 mg/kg oral dosage of CUR in xenograft models, which is equal to the positive control drug, doxorubicin (2 mg/kg) by i.v. administration.

## 1. Introduction

Medicinal plants are gaining interest in the scientific community for health benefits due to their well-known pharmacological action [1,2,3]. The search for anticancer agents from plant sources began in the 1950s [4]. They are a rich and diverse source of chemical constituents with antitumor and cytotoxic properties due to their natural antioxidant and free radical scavenger activity which can reduce or minimize the toxic side effect of chemotherapy [3,5]. The most promising natural herbal extracts with bioactive molecules such as curcumin (turmeric) and artemisinin (qinghao) exhibit pharmacological action, such as antioxidant, antimalarial, antiproliferative, antiangiogenic, anticancer and offer a viable solution to chemotherapeutic drugs [6,7,8].

Curcumin (CUR) is a hydrophobic phytochemical polyphenol of bright yellow color found in the rhizome of turmeric (*Curcuma longa*), chemical diferuloylmethane (C_21_H_20_O_6_). It is generally considered to be the most pharmacologically active constituent of curcuminoids and has many documented activities [8,9]. Artemisinin (ART) is a sesquiterpene lactone natural compound isolated from the plant *Artemesia annua* in 1972 by Youyou Tu (Nobel Prize 2015) [10]. Since 1971, Youyou Tu conducted several antimalarial studies against artemisinin and its derivatives, demonstrated that these compounds exhibit antimalarial activity [11]. Recently, Padmanabhan et al. [12,13] reported synergistic effects of CUR-ART (as a physical mixture) combination having superior antimalarial activity than the individual components. Both compounds CUR and ART have been used in traditional medicine in India and China for over 2000 years. Among CUR and ART natural molecules, ART and its derivatives were approved by FDA as antimalarial drugs in the form of single as well as multidrug therapy with a combination of other drugs [14,15]. However, until today CUR was approved as a nutraceutical but not as a drug [16,17]. Curcumin has poor aqueous solubility (7.8 µg/mL) and low bioavailability (0.051 µg/L) because of rapid metabolism and short half-life [7,18]. Several scientific manuscripts on CUR and ART report potential therapeutic activity as anticancer agents and show cytotoxic activity against a wide range of cancer cell lines including, melanoma, breast, pancreas, and renal cancer cells [19]. Furthermore, xenograft experiments demonstrate the anticancer activity in pancreatic cancer cell lines [20,21]. The insolubility of curcumin makes it a less studied molecule for tumor therapy. In order to enhance its solubility and bioavailability, several formulation strategies have been explored such as polymeric dispersion [22], nanocrystals [23], supramolecular gelators [24], liposomes mixed, suspensions [25]. Our group has reported different solid-state forms of curcumin, i.e., polymorphs [26], cocrystals [27], eutectics [28] and coamorphous [29]. The therapeutic efficacy of both CUR and ART as an amorphous solid with molecular level interactions is not reported.

In previous studies, curcumin-pyrogallol (CUR-PYR) cocrystal exhibited superior dissolution rate compared to other cocrystals as well as eutectics and polymorphs [27]. Subsequently we reported curcumin-artemisinin (CUR-ART) coamorphous solid which showed enhanced bioavailability than pure CUR in male Sprague Dawley (SD) rats at an oral dose of 200 mg/kg. At the same dose, no plasma concentration for curcumin (crystalline) could be detected by HPLC analysis [29] due to its poor aqueous solubility. The present work is a continuation of our previous results on the improved solubility and bioavailability of CUR-PYR cocrystal and CUR-ART coamorphous solid to evaluate the bioavailability of CUR-PYR cocrystals and therapeutic activity of CUR-ART coamorphous solid in xenograft models.

## 2. Materials and Methods

### 2.1. Materials

Curcumin (purity > 99.8%) was obtained from Sigma-Aldrich (Hyderabad, India) and Artemisinin was purchased from Mangalam Drugs (Mumbai, India). Solvents (purity > 99%) were purchased from Merck (Mumbai, India). Water filtered through a double deionized purification system (Aqua DM, Bhanu, Hyderabad, India) was used for all experiments.

### 2.2. Methods

#### 2.2.1. Curcumin Solids Preparation Method

CUR-PYR cocrystal: The cocrystal was obtained by grinding of CUR (1 mmol) and PYR (1 mmol) in 1:1 stoichiometry with a few drops of EtOH added in a liquid-assisted method for 60 min. The resultant product cocrystal was characterized by Powder X-ray diffraction (PXRD) and used in next experiments.

CUR-ART coamorphous: CUR (1 mmol) and ART (1 mmol) were taken in a 1:1 stoichiometric ratio and dissolved in 100 ethanol and rotavaporized at 50–55 °C temperature. The resultant product was characterized and confirmed as a coamorphous solid by PXRD and used in the next experiments.

#### 2.2.2. Characterization of Multicomponent Systems of Curcumin by PXRD

Powder X-ray diffraction (PXRD) was recorded on Bruker D8 Advance diffractometer (Bruker-AXS, Karlsruhe, Germany) using Cu-Kα X-radiation (λ = 1.5406 Å) at 40 kV and 30 mA power. X-ray diffraction patterns were collected over the 2θ range 5–50° at a scan rate of 5°/min.

### 2.3. In Vivo Study Design and Drug Administration

Sprague-Dawley rats (200 ± 50 g) were obtained from Sainath Agencies Limited (Hyderabad, India). Animals were acclimatized for 1 week prior to experimentation in a temperature-controlled, 12/12 h light/dark room, and were allowed standard laboratory food and water. The rats were fasted overnight (~18 h) with free access to water before the experiment. The study was conducted in compliance with standard animal use practices at Virchow Biotech Private Limited, Department of preclinical toxicology, Hyderabad, India (Registration No. 546/02/A/CPSCEA, India). IAEC Approval No. VB/UH/PCT/CUR-ART-2015/PT-3 dated 24-03-2015.

Pharmacokinetic studies of CUR-PYR, CUR-ART physical mixture/coamorphous were conducted in Sprague Dawley rats (male and female) weighing (200 ± 50 g) (*n* = 6) in crossover design. All compounds (200 mg/kg) were suspended in 1% sodium carboxymethylcellulose (CMC) separately and administered by oral gavage. Blood (0.4 mL) was withdrawn from retro-orbital plexus into lithium heparin tube at the following times after drug administration: 30, 45, 60, 90, 120, 180, 240, 360, 480 and 720 min. After centrifugation for 2000 *g* for 15 min, an aliquot of 0.2 mL plasma was collected and extracted curcumin from plasma by deproteination. The resulting plasma was frozen at −80 °C until HPLC analysis.

#### 2.3.1. Determination of Curcumin Plasma Concentration

Curcumin Plasma concentrations of CUR were analyzed by HPLC (Shimadzu, Mumbai, India). The area under the curve (AUC) for serum concentration versus time plots was calculated by linear trapezoidal rate. The maximum plasma concentration, C_max_, and the time T_max_, required to reach C_max_ were obtained from the plasma concentration curve. Shimadzu LC-20AD liquid chromatography.

#### 2.3.2. Chromatographic Conditions

The HPLC analyses were performed using a Shimadzu Prominence model LC-20AD equipped with 20 mL injection loop, and a photodiode array detector and CUR detected at 420 nm. Data acquisition and analysis were carried out using LC solution software (1.25 SP2, Shimadzu, Mumbai, India, 2010). A C18 reversed-phase column (250 mm × 4.6 mm, particle size 5 μm) preceded by a C18 guard column (33 mm × 4.6 mm) was used for analysis. The mobile phase consisted of Acetonitrile- 5% acetic Acid (75:25, *v*/*v*) was run through the column at flow rate of 1.0 mL/min.

### 2.4. Preparation of SGF and SIF Media

Simulated Gastric Fluid (SGF): SGF was prepared according to USP specifications (Test Solutions, United States Pharmacopeia 35, NF 30, 2012). Sodium chloride (0.2 g) was added to a 100 mL flask and dissolved in 50 mL of water. Then 0.7 mL of 10 M HCl was added to adjust the pH of the solution to 1.2. To this, 0.32 g of pepsin was added and dissolved with gentle shaking and the volume made up to 100 mL with water. Pepsin was added only after the pH was adjusted to 1.2.

Simulated Intestinal Fluid (SIF): SIF was prepared according to USP specifications (Test Solutions, United States Pharmacopeia 35, NF 30, 2012). Monobasic potassium phosphate (0.68 g) was dissolved in 25 mL of water, then 7.7 mL of 0.2 N NaOH was added to adjust the pH to 6.8. To this, 1 g of pancreatin was added and shaken gently until clear solution and the volume was adjusted to 100 mL with water. Pancreatin was added after adjusting the pH of the solution to 6.8 to avoid precipitation of the enzyme.

#### Supersaturation Study

Supersaturation of CUR and CUR-ART were measured in SGF and SIF (without enzyme) medium at 30 °C. The supersaturated solution (CUR 100 µg/mL dissolved in 5 mL of SGF/SIF fluid) was stirred at 800 rpm using a magnetic stirrer at 30 °C. After regular intervals of time 2, 4, 6, 12 and 24 h the solution concentration of curcumin was determined at 420 nm maxima on a Thermo Scientific Evolution 300 UV-Vis spectrometer (Thermo Scientific, Waltham, MA, USA).

### 2.5. Acute Toxicity Study

In two different acute toxicity studies, Sprague Dawley (SD) and Swiss albino mice (female and male) were used and obtained from Sainath Agencies Limited (Hyderabad, India). The study was conducted as per norms of the institutional guideline, quality assurance officer (QAO) with animal welfare regulations under an approved protocol by the Institutional Animal Care and Use Committee at Virchow Biotech Private Limited, Department of preclinical toxicology, Hyderabad, India (Registration No. 546/02/A/CPSCEA, India).

In two acute studies, Sprague Dawley (SD) rats (6M + 6F/group: 6–8 weeks of age; body weight range: 180–220 g) and Swiss albino mice (6M + 6F/group; 6–8 weeks of age; body weight range: 18–21 g) were administered a single oral (gavage) dose of CUR-ART coamorphous and CUR-PYR cocrystal suspended in sodium carboxymethyl cellulose (CMC) at levels of 0 and 2000 mg/kg body weight. In all these experiments, animals were observed for 15 days for clinical signs as well as for morbidity and mortality. On day 15 at completion, all the animals were euthanized and gross pathological examinations were undertaken.

### 2.6. In Vitro Cell Culture

The PANC-1 cells derived from human pancreatic carcinoma of ductal cell origin were obtained from National Center for Cell Science, Pune, India. The cells were cultured in Dulbecco’s modified medium (DMEM) supplemented with 10% *v*/*v* fetal bovine serum (FBS), 1% penicillin, and 1% streptomycin. Cultures (Figure S1, Supplementary Materials) were maintained at 37 °C in a humidified atmosphere with 5% CO_2_.

### 2.7. In Vivo PANC-1 Tumor Growth Xenograft Model

Female athymic nude mice were obtained from the National Centre for Laboratory Animal Sciences (NCLAS), National Institute of Nutrition (ICMR), Hyderabad, India and were selected after careful initial screening for any external signs of disease or injuries. They were housed in individual cages in the conventional animal facilities of NCLAS, a registered facility with Committee for the Purpose of Control and Supervision of Experimentation on Animals (CPCSEA) (154/1999), Government of India. The environmental conditions were kept at 21 ± 2 °C, with 10–15 air changes per hour and relative humidity of 50–55 percent with 12 h light/dark cycle water. Animals were quarantined for 4 days and all the animals had free access to sterile formulated feed pellets and filtered potable clean water.

PANC-1 cells (1 × 10^6^) using matrigel were injected subcutaneously into the flanks of mice (Figure S2, Supplementary Materials). Tumor-bearing mice were then divided randomly into six treatment groups (six mice per group) and treatment initiated when the xenografted solid tumors reached a volume of about 80–100 mm^3^. Each mouse was administered orally (p.o.) every day with either control vehicle (CMC), CUR (50 mg/kg), ART (50 mg/kg), CUR-ART (50 and 100 mg/kg) and Doxorubicin (2 mg/kg) by intravenous (i.v.) route. All mice were cared for and maintained in accordance with animal welfare regulations under an approved protocol by the Institutional Animal Care and Use Committee at Virchow Biotech Private Limited, Department of preclinical toxicology, Hyderabad, India (Registration No. 546/02/A/CPSCEA, India).

After xenograft transplantation, mice exhibiting tumors were monitored (Figure S3, Supplementary Materials) and tumor size was measured once every 3 and 5 days using caliper. The tumor volume in each animal was estimated according to the formula: tumor volume (mm^3^) = *L* × *W*^2^/2 (where *L* is the length and *W* are the widths) with the final measurement taken 4 weeks after tumor cell inoculation. At the same time, the body weight of each animal was measured. At the end of the experiment (4 weeks after cell inoculation), the animals were euthanized by CO_2_ and sacrificed. Tumors from each animal were removed and measured.

### 2.8. Statistical Analysis

#### 2.8.1. Bioavailability Study

The standard curve was calculated by a linear relationship between concentration and area with regression factor R = 0.999. The Area under the curve (AUC) for serum concentration vs. Time plots was calculated by the linear trapezoidal rule. The maximum plasma concentration C_max_ and the time T_max_ required to reach C_max_ were obtained from the plasma concentration curve. Statistical analysis was carried out, where matched paired *t*-test comparison and repeated measures of ANOVA were applied for different time points.

#### 2.8.2. Xenograft Studies

Each value represents mean ± SD (Standard Deviation). The control and experimental animal groups were compared by paired *t*-test. **** *p* < 0.0001 was considered significant.

## 3. Results and Discussion

**Scheme 1:**
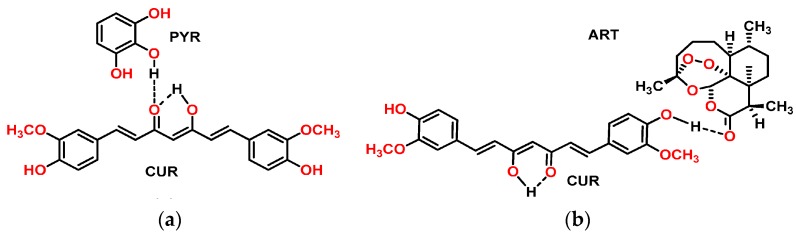
The structure of Curcumin-Pyrogallol (CUR-PYR) cocrystal (**a**) and Curcumin-Artemisinin (CUR-ART) co-amorphous (**b**) are stabilized through intermolecular O–H···O hydrogen bonding.

**Figure 1:**
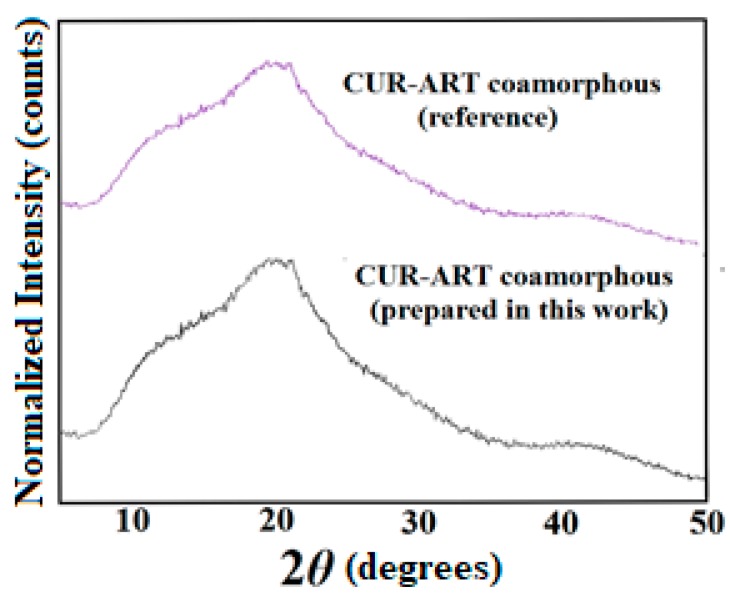
Bulk phase of CUR-ART powder X-ray diffraction (PXRD) pattern matches with the reported reference compound by PXRD [29].

**Figure 2:**
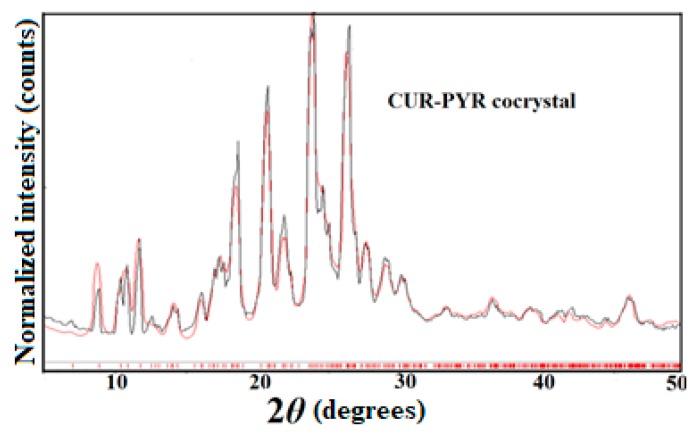
Experimental PXRD (black trace) of CUR-PYR match nicely with the calculated X-ray diffraction lines from the crystal structure (red), indicating the excellent purity of the bulk phase [27].

CUR-PYR cocrystal and CUR-ART coamorphous solids are associated through intermolecular O–H···O hydrogen bonding (Scheme 1). The crystal structure of CUR-PYR was determined by single crystal X-ray diffraction and the hydrogen bonding synthons are known [28]. Hydrogen bonding in CUR-ART is inferred from IR (Infrared) spectra and preferred hydrogen bonding interaction of strongest donor-strongest acceptor in the crystal structure (in the absence of X-ray data for the amorphous solid) [29]. The bulk phase composition was produced by co-grinding (CUR-PYR) and by fast evaporation under vacuum (CUR-ART) as detailed in the Experimental Section. The bulk phase composition was prepared reproducibly and characterized by powder X-ray diffraction pattern (Figure 1 and Figure 2). The same materials were used in the pharmacokinetic, phase stability, toxicology and xenograft studies.

### 3.1. Bioavailability Studies

**Figure 3:**
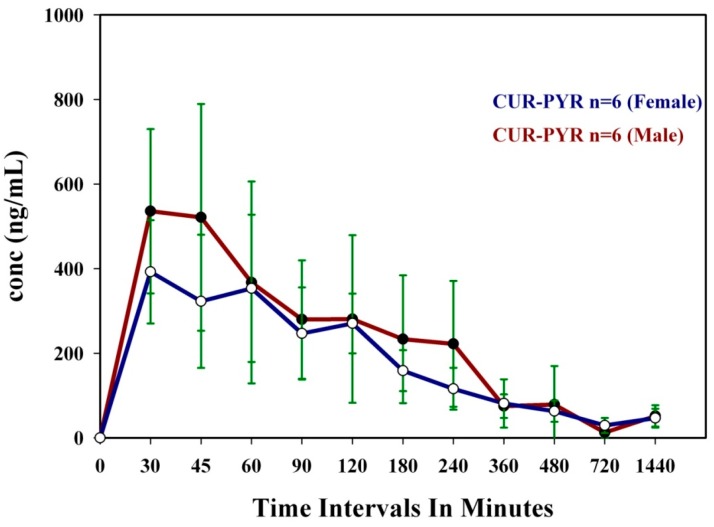
Mean Plasma concentration of cocrystal CUR-PYR vs. Time profile obtained from both female and male Sprague Dawley (SD) rats.

**Figure 4:**
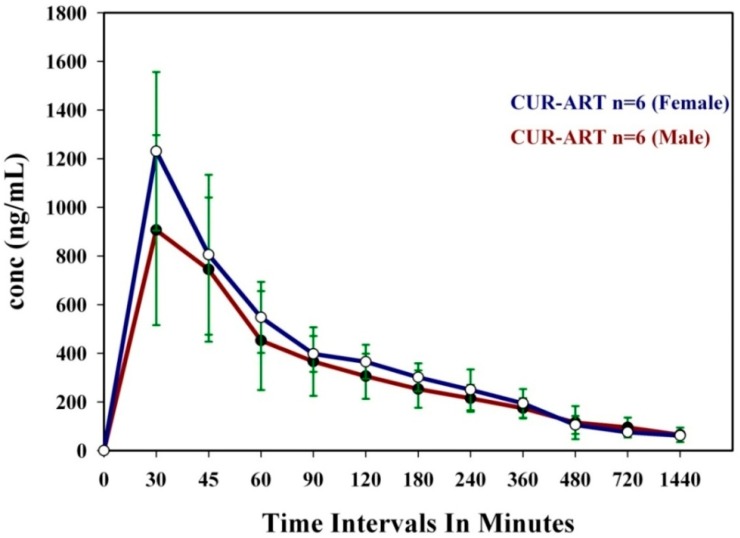
Mean Plasma concentration of CUR-ART coamorphous vs. Time profile obtained from both female and male SD rats.

Oral bioavailability is the most important property for drug delivery. Over 80% of drugs are marketed worldwide as tablets and capsules. Oral bioavailability is the fraction of the solid dose administered which reaches systemic circulation in blood/plasma. The physicochemical properties of an API or bioactive molecule can be enhanced to give higher solubility and faster dissolution rate through salts, amorphous, cocrystal or coamorphous forms with better absorption and improved bioavailability. With the objective to improve the systemic bioavailability of CUR [30,31] in circulation, we have taken forward the superior dissolution rates of CUR-PYR cocrystal and CUR-ART coamorphous as leads in animal studies. The cocrystal and coamorphous phases of curcumin with a dose of 200 mg/kg (of active curcumin) were administered orally in SD rats (both male and female) to monitor the systemic bioavailability of circulating curcumin. The results of cocrystal and coamorphous solids mean concentrations in the serum are illustrated in Table 1, and Figure 3 and Figure 4. In female SD rats, CUR-ART coamorphous exhibited superior serum levels of 1.23 μg/mL at 30 min and 0.43 μg/mL of CUR-PYR cocrystal at 45 min post administration, whereas in male rats the CUR-ART value is 0.90 μg/mL and CUR-PYR is 0.53 μg/mL at 30 min respectively. The apparent half-life (T_1/2_) increased significantly to 6–7 h for both CUR-PYR and CUR-ART, which is much longer than that of pure curcumin (<1 h). CUR-ART coamorphous exhibited approximately two-fold superior bioavailability compared to CUR-PYR cocrystal. It is difficult to compare the enhancement with pure curcumin because the soluble fraction is too low to be detected by HPLC method. Sasaki et al. have reported theracurcumin (colloidal dispersion with gum-gatti) [32] formulation at 300 mg/kg oral dosage with high C_max_ value of 1.69 µg/mL at T_max_ of 120 min and amount of CUR concentration delivered in vivo of AUC_(0–∞)_ 9.3 mg·h/mL. CUR-ART coamorphous (at 200 mg/kg dose) exhibited high C_max_ of 0.9–1.2 µg/mL (which is comparable to theracurcumin) at short T_max_ (30 min), meaning immediate drug delivery. Specifically AUC_(0–∞)_ 35 mg·h/mL for CUR-ART coamorphous solid is 3.7 times greater than that for theracurcumin [32]. This is a remarkable improvement in the conc. of the CUR at short T_max_ of 30 min and total drug delivered AUC_(0–∞)_. Oral administration of pure CUR and CUR-ART physical mixture did not show any detectable levels in the serum attributable to CUR very low solubility and short half-life. This observation is consistent with recent reports wherein pure curcumin could not be detected at even higher dosage levels (>1 g/kg) by HPLC. To summarize, the C_max_ for CUR-PYR curves do not peak so sharply and rapidly as that for CUR-ART. This is attributed to the coamorphous phase solid-state form of CUR-ART compared to the crystalline state of CUR-PYR. The high free energy of amorphous solids used imparts higher solubility and bioavailability due to high thermodynamic functions.

### 3.2. Phase Stability in SGF and SIF Media

**Figure 5:**
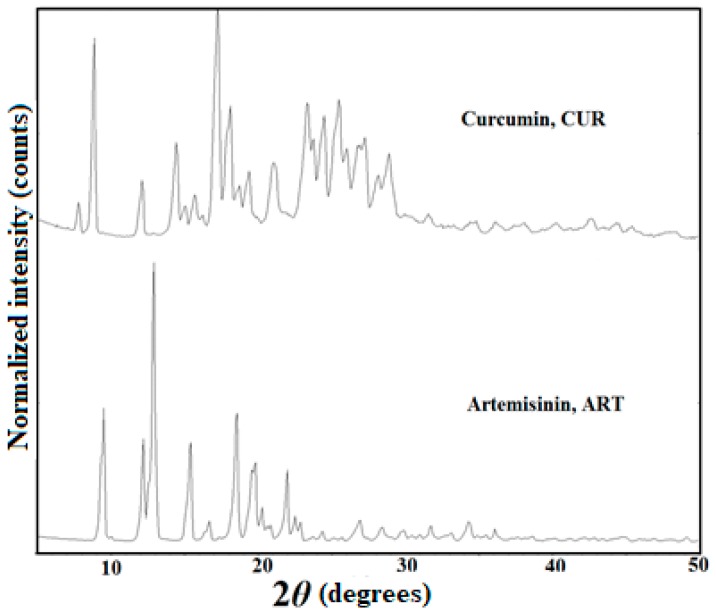
PXRD patterns of starting materials CUR (form 1) and ART (form 1).

Typically amorphous solids exhibit rapid dissolution rate and high bioavailability in plasma. CUR-ART coamorphous displayed a short T_max_ but prolonged half-life in vivo (6–7 h). The latter result is significant because the short half-life of pure curcumin (45–60 min) in crystalline form severely limits the active drug delivered. We reasoned that CUR-ART coamorphous is stabilized as dispersion compared to pure curcumin. In vitro slurry experiments in SGF and SIF media (simulated gastric fluid and simulated intestinal fluid of pH 1.2 and 7, respectively) were performed to understand the stability of the amorphous phase by powder X-ray diffraction. PXRD of the reference compound stable form are shown in Figure 5. In addition, we also conducted supersaturation experiments to find out the soluble curcumin precipitation in both CUR (crystalline) and CUR-ART coamorphous forms.

**Figure 6:**
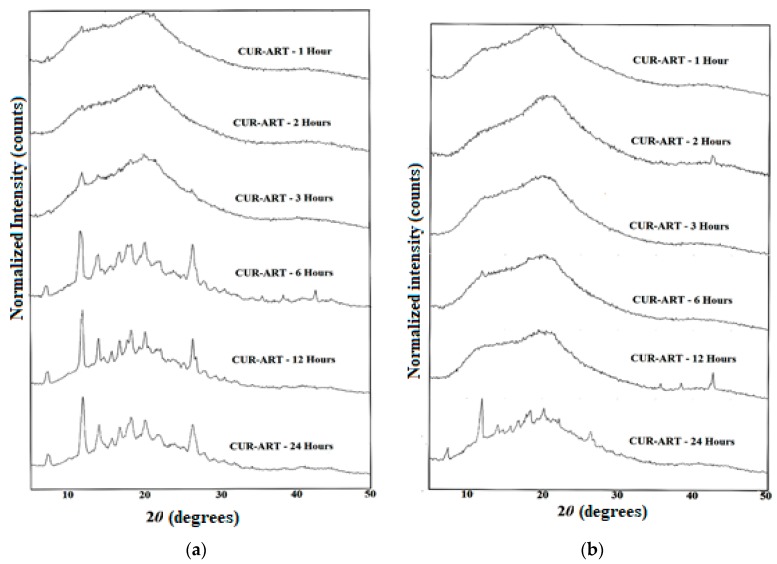
CUR-ART phase stability study PXRD patterns at different time intervals in Simulated Gastric Fluid (SGF), pH = 1.2 (**a**); and Simulated Intestinal Fluid (SIF) media, pH = 7 (**b**). The precipitated polymorph of ART is form II.

**Figure 7:**
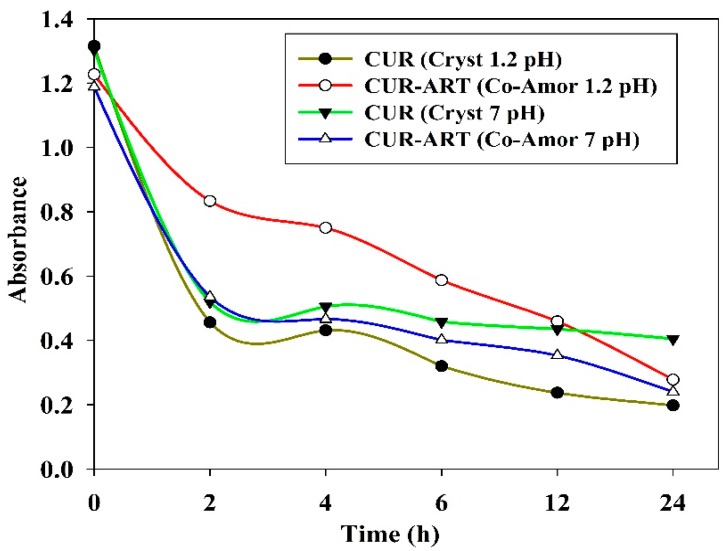
Supersaturation experiments of CUR and CUR-ART: UV-Vis plots of Absorbance vs. Time recorded at 420 nm peak intensity for curcumin. Note the higher concentration of curcumin from CUR-ART in acidic medium (brown trace), which indicates supersaturation in the gastric environment.

**Figure 8:**
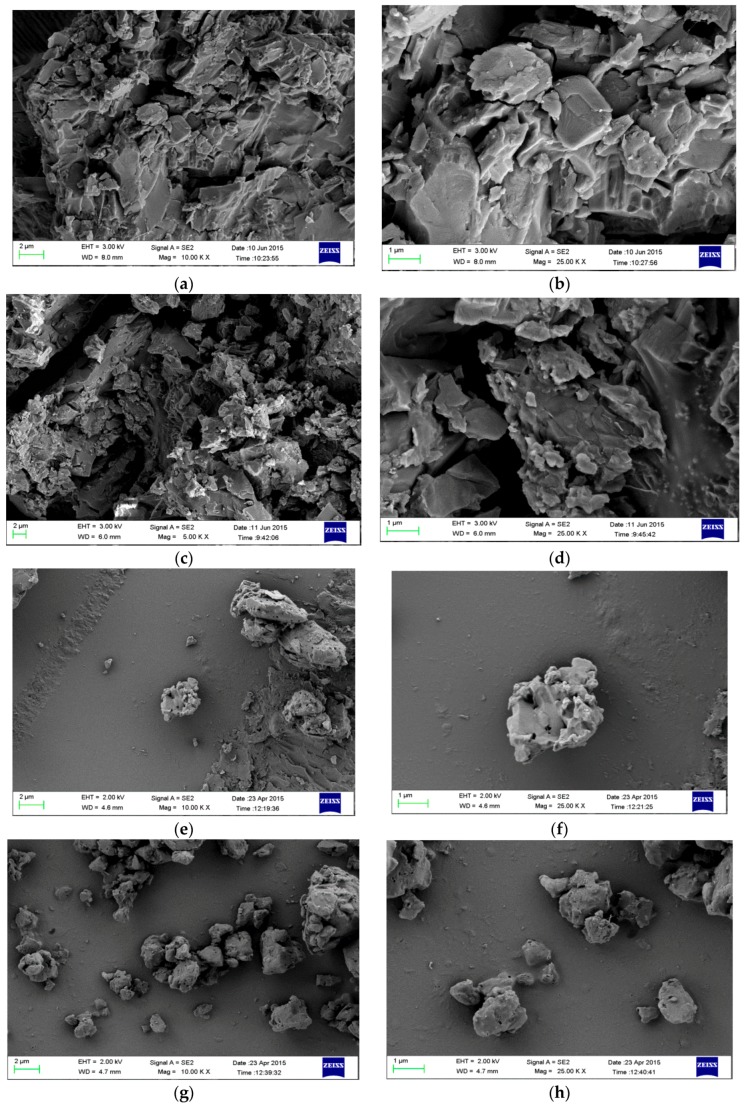
CUR-ART in SGF/SIF at 2 µm and 1 µm resolution. (**a**,**b**) are slurry material in SGF at 1 h; (**c**,**d**) are slurry material in SGF at 2 h; (**e**,**f**) are slurry material in SGF at 24 h; (**g**,**h**) are slurry material in SIF at 24 h. The agglomerates of larger particles up to 2 h time point have separated/dissociated to give smaller particles by 24 h.

**Scheme 2:**
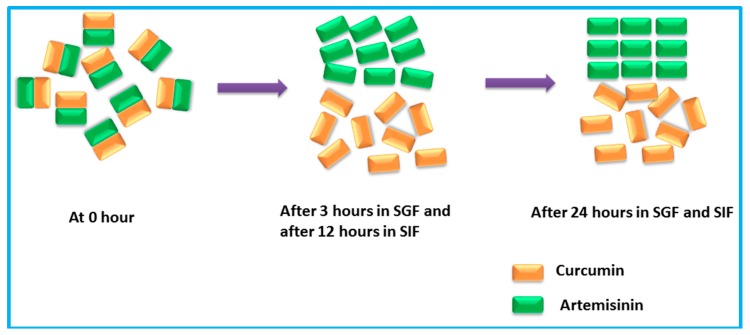
Pictorial representation of CUR-ART physical stability in simulated fluids.

CUR-ART solid physical stability were conducted in the supersaturated solution of SGF/SIF media at 1000 rpm using magnetic stirrer at 30 °C for different time intervals (1, 2, 3, 6, 12 and 24 h) in separate experiments. At the end point, the suspension was filtered and the solid residue tested by powder X-ray diffraction. CUR-ART coamorphous was noted to be stable for up to 3 h in SGF medium. After about 6 h it dissociated and the ART component precipitated as the crystalline form (ART form II precipitated) [34] but CUR component was still present in amorphous/semi crystalline state as observed by PXRD (Figure 6a). Similarly, in SIF medium CUR-ART coamorphous was stable for up to 12 h (Figure 6b) and after 12 h the ART component precipitated in crystalline state. These results suggest a reason for the high solubility of curcumin in CUR-ART. The amorphous form of CUR is present for a long enough time in the simulated conditions to release high drug concentration over an extended period of time (several hours). This explains the longer half-life (up to 6–7 h) of curcumin in SD rats when administered as CUR-ART coamorphous. In contrast, pure curcumin decomposes in less than 1 h. The extended half-life of curcumin which dissociates slowly from CUR-ART is due to the strong intermolecular O–H···O hydrogen bonds in the structure. The phase stability of CUR-ART in SGF and SIG media and its high solubility are explained in Scheme 2 as the “spring and parachute” model of cocrystal solubility for drugs [31]. The mechanism of Scheme 2 for CUR-ART was supported by supersaturation experiments of CUR and CUR-ART in SGF and SIF media (without enzyme) and analyzed by UV spectroscopy (Figure 7). From these experiments we observed that CUR precipitated faster than CUR-ART (Figure 8) at 100 µg/mL concentration of CUR. An additional UV maxima (at 360 nm) was observed in SIF medium, due to degradation of CUR since it is known that curcumin is less stable as the pH increases. These experiments indicate that solubilized CUR in CUR-ART has longer residence time than pure CUR in both SGF and SIF, which is due to noncovalent interactions between CUR and ART in the coamorphous solid.

The particle morphology at time point 1, 2 and 24 h of the precipitated CUR-ART coamorphous was analyzed by FESEM (Field Emission Scanning Electron Microscope). The high magnification images clearly show that the large particles are actually agglomerates of smaller particles at 1 h and 2 h in SGF medium. There are irregular, densely shape particles in both pH media by 24 h (Figure 8).

### 3.3. Acute Toxicity Study

In the acute toxicity studies, a high dose of 2000 mg/kg (active curcumin) of CUR-ART coamorphous and CUR-PYR cocrystal was tested in Swiss albino mice and SD rats. During the 15 days observation period of acute oral toxicity and body weight measurements, no toxic were observed in either of the two species (data are shown in Tables S1–S4, Supplementary Materials). No mortality or morbidity was found in the treated group at the end of the study and no gross pathological abnormalities were observed in rats or mice. Physical, physiological and neurological parameters assessed in the exposed animals were in the normal range and well tolerated without any adverse systemic toxicity. Thus, curcumin-artemisinin is safe at the tested dose and sub-chronic toxicity may be evaluated at different strengths.

### 3.4. In Vivo Antitumor Effect of CUR-ART against Panc-1 Xenograft

**Figure 9:**
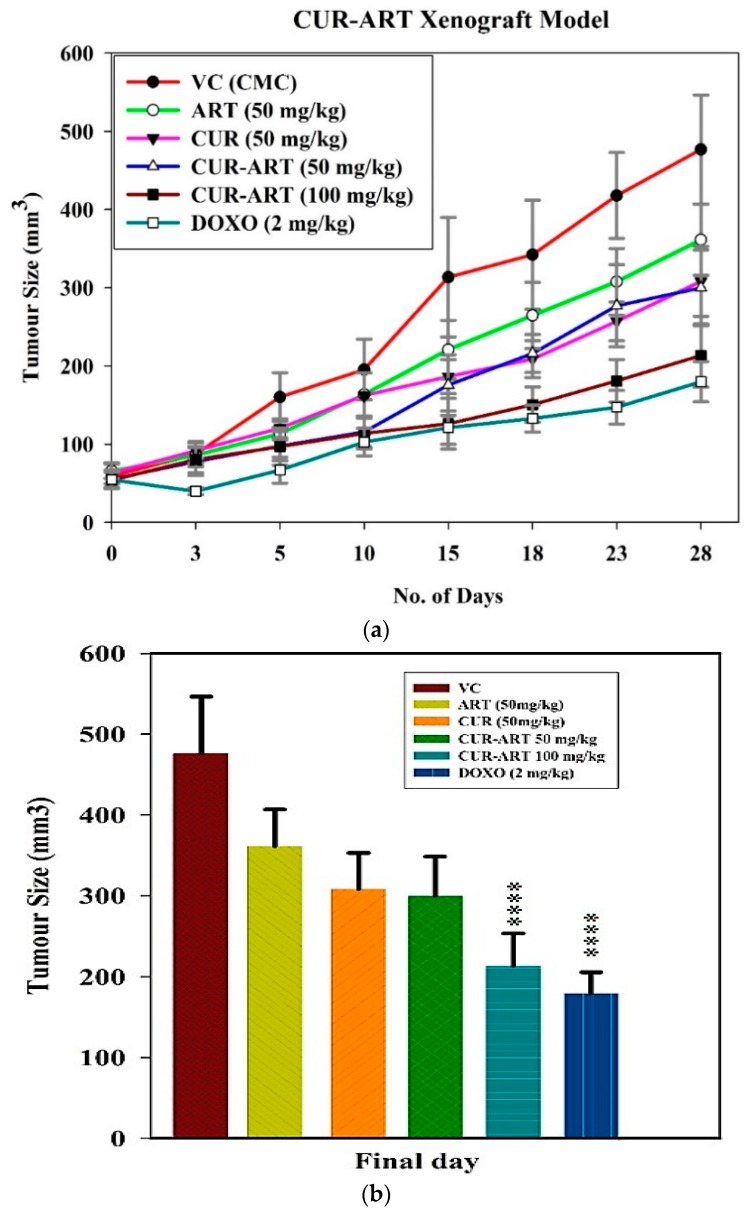
(**a**) Effect of vehicle control (VC), ART, CUR, CUR-ART and doxorubicin (DOXO) on tumor size during the study period; (**b**) Comparison of tumor size on day 28.

**Figure 10:**
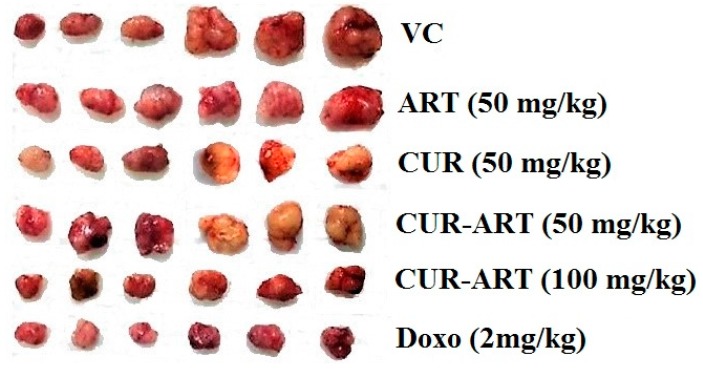
Tumor volume of Panc-1 xenograft model in nude mice during therapy under different drug treatments.

With enhanced bioavailability, improved stability and low toxicity of CUR-ART, the coamorphous solids was tested in xenograft animal study (*n* = 7) to know its therapeutic efficacy. This study was investigated in the induced pancreas xenograft (tumor was developed by Panc-1 cells) model in nude mice. The compounds were administered systemically with CUR-ART (50 and 100 mg/kg) coamorphous relative to the control (vehicle control, VC, 1% CMC, sodium carboxymethyl cellulose), CUR (50 mg/kg), ART (50 mg/kg), (CUR-ART 50 and 100 mg/kg) and doxorubicin (DOXO, 2 mg/kg). Doxorubicin, a standard drug for pancreatic cancer, was used as positive control. CUR-ART, CUR and ART were administered orally with 1% CMC solution while doxorubicin was delivered through the tail vein (i.v.). All drugs were administered daily for 5 weeks. A schematic representation of the six groups employed in this study (Figure 9a) shows that CUR-ART coamorphous and doxorubicin decreased tumor size significantly (*p* < 0.0001) compared to all other solids and controls. The percentage of inhibition of CUR-ART (100 mg/kg) is 61.87% which is close to doxorubicin 69.97% (2 mg/kg). The inhibition in other treated groups (at 50 mg/kg) is ART 29.22%, CUR 41.23% and CUR-ART coamorphous 41.41%. Based on these in vivo experiments, it is clear that CUR-ART coamorphous can be an effective treatment to inhibit tumor growth in Panc-1 xenograft mice model at 100 mg/kg dose. On day 28, the experimental mice were euthanized and tumors were excised from each group. The picture of these dissected tumors shows that the size of the tumor treated with 100 mg/kg CUR-ART coamorphous and 2 mg/kg doxorubicin is the smallest among other treated and non-treated groups (Figure 9b and Figure 10). The body weight was similar in all the treated and non-treated groups (Figure S4, Supplementary Materials). Significant inhibition of tumor growth was evident in nude mice by treatment with CUR-ART coamorphous (p.o.) and Doxorubicin (i.v.) at 100 mg/kg/day and 2 mg/kg/day respectively (Figure 10). This indicates that solo or combination therapies could be considered to achieve fast treatment of cancer with minimal side effects.

## 4. Conclusions

Examples of coamorphous drugs are as such rare in the literature [35,36,37,38]. Curcumin and artemisinin are herbal drugs of Indian and Chinese origins. The low solubility and poor bioavailability of curcumin pose a limitation to making drug formulation of curcumin. We have overcome the multiple disadvantages of curcumin in the designed CUR-ART coamorphous dispersion. This novel binary solid exhibits high solubility and bioavailability, extended half-life and action of bioactive curcumin at normal drug dose regime of 100 mg/kg. The therapeutic activity of CUR-ART in Xenograft models of Panc-1 is comparable to commercial drug doxorubicin. The diverse biological action of curcumin and artemisinin, and the high dose of net drug delivered as coamorphous solid along with possible synergistic effects could open the opportunity for a herbal formulation to treat cancer and malaria.

## Figures and Tables

**Table 1:** Pharmacokinetic data of Curcumin-Pyrogallol (CUR-PYR) cocrystal vs. Curcumin-Artemisinin (CUR-ART) coamorphous solids ^a^.

Parameters	CUR-PYR (F)		CUR-ART (F)		CUR-PYR (M)		CUR-ART (M)	
	Mean	S.D.	Mean	S.D.	Mean	S.D.	Mean	S.D.
T_max_ (min)	45.00	15.60	30.00	0.00	30.00	8.22	30.00	0.00
C_max_ (µg/mL)	0.43	0.15	1.23	0.33	0.53	0.19	0.90	0.39
T_1/2_ (h)	7.70	3.27	6.70	0.68	6.00	2.78	7.00	1.66
AUC_(0–24)_ (µg·h/mL)	1.91	0.25	3.69	0.69	2.18	0.59	3.45	0.83
AUC_(0–∞)_ (µg·h/mL)	22.20	6.70	36.40	6.00	19.70	5.72	35.40	5.64

^a^ Unformulated curcumin at 340 mg/kg oral dosage: T_max_ 30 min, C_max_ 0.0024 µg/mL, AUC 4.8 µg·min/mL [33]; AUC: the area under the curve; S.D.: standard deviation; T_max_: the maximum plasma time; C_max_: the maximum plasma concentration; T_1/2_: the apparent half-life.

## Supplementary Materials

The following are available online at www.mdpi.com/1999-4923/10/1/7/s1, Figure S1: Morphology of PANC-1 cells; Figure S2: Implantation of cells through subcutaneous route (S.C.) using Matrigel; Figure S3: Tumor development of PANC-1 implanted cell lines in mice; Figure S4: Body weight of selected nude mice for the xenograft studies; Table S1: Acute oral toxicity of CUR-PYR cocrystal in Sprague Dawley Rats (Male and Female); Table S2: Acute oral toxicity of CUR-ART coamorphous in Sprague Dawley Rats (Male and Female); Table S3: Acute oral toxicity of CUR-ART coamorphous in Swiss Albino Mice (Male and Female); Table S4: Acute oral toxicity of CUR-PYR cocrystal in Swiss Albino Mice (Male and Female).

Supplementary File 1
