# Supplementary material for: Curcumin-Artemisinin Coamorphous Solid: Xenograft Model Preclinical Study
Source: Pharmaceutics. 2018 Jan 9;10(1):7. doi: 10.3390/pharmaceutics10010007 (PMC5874820; doi:10.3390/pharmaceutics10010007)
Supplement: Supplementary File 1 [file pharmaceutics-10-00007-s001.pdf]

Curcumin-Artemisinin Coamorphous Solid: Xenograft Model Preclinical Study

M. K. Chaitanya Mannava [1,2], Kuthuru Suresh [1,2], Manish Kumar Bommaka [1], Durga Bhavani Konga [4] and Ashwini Nangia [1,2,3,*]

[1] School of Chemistry, University of Hyderabad, Hyderabad 500 046, India; mchaitanyasharma@gmail.com (M.K.C.M.); kuturusuresh@gmail.com (K.S.); mani071115@gmail.com (M.K.B.)
[2] Technology Business Incubator, University of Hyderabad, Hyderabad 500 046, India
[3] Council of Scientific and Industrial Research National Chemical Laboratory, Pune 411 008, India
[4] Virchow Biotech Pvt. Ltd., Animal House Facility, Hyderabad 500 055, India; kongabilwakshi@gmail.com
* Correspondence: ashwini.nangia@gmail.com or ak.nangia@ncl.res.in; Tel.: +91-202-590-2600

Table S1. Acute oral toxicity of CUR-PYR cocrystal in Sprague Dawley Rats (Male and Female).

Body weights (g)—Male

Groups	Day 0	Day 4	Day 8	Day 12	Day 15
VC	210.68 ± 4.15 (6)	213 ± 3.88 (6)	216.16 ± 3.79 (6)	219.25 ± 3.37 (6)	220.66 ± 3.26 (6)
TG	209.16 ± 4.03 (6)	211.83 ± 1.91 (6)	215.58 ± 4.97 (6)	219.08 ± 5.31 (6)	219.83 ±5.24 (6)

Values are expressed in Mean ± S.D () No. of Animals
VC-Vehicle Control
TG-Theraupatic Group

Body weights (g)—Female

Groups	Day 0	Day 4	Day 8	Day 12	Day 15
VC	206.91 ± 3.92 (6)	210.5 ± 3.84 (6)	214.58 ± 3.74 (6)	218.0 ± 3.68 (6)	218.73 ± 3.94 (6)
TG	206.7 ± 3.34 (6)	210.16 ± 3.43 (6)	213.91 ± 3.07 (6)	216.48 ± 2.96 (6)	218.83 ± 2.90 (6)

Values are expressed in Mean ± S.D () No. of Animals
VC-Vehicle Control
TG-Theraupatic Group

Organ Weights (g)—Male

Groups	Heart	Lung	Liver	Kidney	Spleen	Brain	Testis
VC	0.86 ± 0.03 (6)	1.17 ± 0.05 (6)	9.20 ± 0.05 (6)	1.44 ± 0.01 (6)	0.84 ± 0.16 (6)	1.90 ± 0.04 (6)	1.76 ± 0.07 (6)
TG	0.87 ± 0.06 (6)	1.14 ± 0.01 (6)	9.10 ± 0.12 (6)	1.39 ± 0.03 (6)	0.83 ± 0.18 (6)	1.89 ± 0.03 (6)	1.73 ± 0.11 (6)

Values are expressed in Mean ± S.D () No. of Animals
VC-Vehicle Control
TG-Theraupatic Group

Organ Weights (g)—Female

Groups	Heart	Lung	Liver	Kidney	Spleen	Brain	Ovaries
VC	0.80 ± 0.04 (6)	1.11 ± 0.06 (6)	7.40 ± 0.09 (6)	1.33 ± 0.05 (6)	0.54 ± 0.03 (6)	1.83 ± 0.06 (6)	0.05 ± 0.02 (6)
TG	0.82 ± 0.02 (6)	1.10 ± 0.03 (6)	7.43 ± 0.7 (6)	1.13 ± 0.07 (6)	0.56 ± 0.08 (6)	1.82 ± 0.05 (6)	0.06 ± 0.02 (6)

Values are expressed in Mean ± S.D () No. of Animals
VC-Vehicle Control
TG-Theraupatic Group

Table S2. Acute oral toxicity of CUR-ART coamorphous in Sprague Dawley Rats (Male and Female).

Body weights (g)—Male

Groups	Day 0	Day 4	Day 8	Day 12	Day 15
VC	215.61 ± 2.8 (6)	207.08 ± 2.85 (6)	209.83 ± 3.98 (6)	213.0 ± 2.75 (6)	214.41 ± 2.51 (6)
TG	214.7 ± 2.43 (6)	216.16 ± 2.18 (6)	218.5 ± 2.07 (6)	220.75 ± 1.69 (6)	221.83 ± 1.47 (6)

Values are expressed in Mean ± S.D () No. of Animals
VC-Vehicle Control
TG-Theraupatic Group

Body weights (g)—Female

Groups	Day 0	Day 4	Day 8	Day 12	Day 15
VC	185.85 ± 2.28 (6)	187.0 ± 2.54 (6)	190.91 ± 1.88 (6)	193.28 ± 1.36 (6)	196.58 ± 1.02 (6)
TG	195.65 ± 3.18 (6)	197.83 ± 2.65 (6)	201.5 ± 2.88 (6)	204.51 ± 1.62 (6)	206.35 ± 2.06 (6)

Values are expressed in Mean ± S.D () No. of Animals
VC-Vehicle Control
TG-Theraupatic Group

Organ Weights (g) —Male

Groups	Heart	Lung	Liver	Kidney	Spleen	Brain	Testis
VC	0.82 ± 0.09 (6)	1.15 ± 0.01 (6)	9.12 ± 0.01 (6)	1.42 ± 0.07 (6)	0.83 ± 0.05 (6)	1.95 ± 0.03 (6)	1.75 ± 0.09 (6)
TG	0.87 ± 0.06 (6)	1.14 ± 0.01 (6)	9.10 ± 0.12 (6)	1.39 ± 0.03 (6)	0.83 ± 0.18 (6)	1.89 ± 0.03 (6)	1.73 ± 0.11 (6)

Values are expressed in Mean ± S.D () No. of Animals
VC-Vehicle Control
TG-Theraupatic Group

Organ Weights (g)—Female

Groups	Heart	Lung	Liver	Kidney	Spleen	Brain	Ovaries
VC	0.82 ± 0.08 (6)	1.12 ± 0.13 (6)	7.50 ± 0.07 (6)	1.30 v 0.01 (6)	0.61 ± 0.07 (6)	1.88 ± 0.017 (6)	0.07 ± 0.01 (6)
TG	0.81 ± 0.02 (6)	1.05 ± 0.06 (6)	7.56 ± 0.4 (6)	1.28 ± 0.09 (6)	0.59 ± 0.07 (6)	1.87 ± 0.015 (6)	0.05 ± 0.01 (6)

Values are expressed in Mean ± S.D () No. of Animals
VC-Vehicle Control
TG-Theraupatic Group

Table S3. Acute oral toxicity of CUR-ART coamorphous in Swiss Albino Mice (Male and Female).

Body weights (g)—Male

Groups	Day 0	Day 4	Day 8	Day 12	Day 15
VC	18.8 ± 0.4 (6)	19.75 ± 0.52 (6)	21.4 ± 0.37 (6)	24.0 ± 0.63 (6)	25.58 ± 0.58 (6)
TG	20.81 ± 0.9 (6)	21.7 ± 0.89 (6)	23.41 ± 0.37 (6)	24.66 ± 0.25 (6)	26.43 ± 0.38 (6)

Values are expressed in Mean ± S.D () No. of Animals
VC-Vehicle Control
TG-Theraupatic Group

Body weights (g)—Female

Groups	Day 0	Day 4	Day 8	Day 12	Day 15

VC	17.83 ± 0.12 (6)	19.0 ± 0.44 (6)	19.9 ± 0.49 (6)	21.91 ± 0.49 (6)	23.5 ± 0.44 (6)
TG	19.03 ± 0.50 (6)	20.41 ± 0.37 (6)	22.16 ± 0.40 (6)	23.83 ± 0.25 (6)	24.91 ± 0.73 (6)

Values are expressed in Mean ± S.D () No. of Animals
VC-Vehicle Control
TG-Theraupatic Group

Organ Weights (g)—Male

Groups	Heart	Lung	Liver	Kidney	Spleen	Brain	Testis
VC	0.14 ± 0.05 (6)	0.41 ± 0.07 (6)	1.3 ± 0.04 (6)	0.4 ± 0.05 (6)	0.06 ± 0.01 (6)	0.4 ± 0.005 (6)	0.22 ± 0.03 (6)
TG	0.14 ± 0.01 (6)	0.34 ± 0.06 (6)	1.30 ± 0.03 (6)	0.49 ± 0.05 (6)	0.061 ± 0.01 (6)	0.47 ± 0.005 (6)	0.22 ± 0.02 (6)

Values are expressed in Mean ± S.D () No. of Animals
VC-Vehicle Control
TG-Theraupatic Group

Organ Weights (g)—Female

Groups	Heart	Lung	Liver	Kidney	Spleen	Brain	Ovaries
VC	0.11 ± 0.013 (6)	0.30 ± 0.07 (6)	1.03 ± 0.08 (6)	0.30 v 0.026 (6)	0.07 ± 0.002 (6)	0.47 ± 0.06 (6)	0.05 ± 0.01 (6)
TG	0.11 ± 0.012 (6)	0.32 ± 0.05 (6)	1.06 ± 0.04 (6)	0.38 ± 0.022 (6)	0.07 ± 0.002 (6)	0.45 ± 0.08 (6)	0.02 ± 0.01 (6)

Values are expressed in Mean ± S.D () No. of Animals
VC-Vehicle Control
TG-Theraupatic Group

Table S4. Acute oral toxicity of CUR-PYR cocrystal in Swiss Albino Mice (Male and Female).

Body weights (g)—Male

Groups	Day 0	Day 4	Day 8	Day 12	Day 15
VC	18.93 ± 1.57 (6)	20.5 ± 1.94 (6)	23.08 ± 2.17 (6)	25.41 ± 2.51 (6)	25.38 ± 3.24 (6)
TG	19.23 ± 1.70 (6)	21.11 ± 1.35 (6)	23.73 ± 1.32 (6)	26.25 ± 1.89 (6)	26.4 ± 1.41 (6)

Values are expressed in Mean ± S.D () No. of Animals
VC-Vehicle Control
TG-Theraupatic Group

Body weights (g)—Female

Groups	Day 0	Day 4	Day 8	Day 12	Day 15
VC	20.0 ± 1.44 (6)	21.41 ± 1.15 (6)	23.65 ± 1.14 (6)	25.75 ± 1.3 (6)	26.11 ± 1.70 (6)
TG	18.75 ± 1.44 (6)	20.58 ± 1.90 (6)	22.83 ± 1.63 (6)	25.0 ± 1.34 (6)	25.25 ± 1.80 (6)

Values are expressed in Mean ± S.D () No. of Animals
VC-Vehicle Control
TG-Theraupatic Group

Organ Weights (g)—Male

Groups	Heart	Lung	Liver	Kidney	Spleen	Brain	Testis
VC	0.14 ± 0.06 (6)	0.42 ± 0.06 (6)	1.40 ± 0.07 (6)	0.41 ± 0.07 (6)	0.05 ± 0.02 (6)	0.52 ± 0.06 (6)	0.27 ± 0.02 (6)
TG	0.15 ± 0.03 (6)	0.44 ± 0.03 (6)	1.41 ± 0.05 (6)	0.43 ± 0.03 (6)	0.06 ± 0.02 (6)	0.52 ± 0.004 (6)	0.25 ± 0.03 (6)

Values are expressed in Mean ± S.D () No. of Animals
VC-Vehicle Control
TG-Theraupatic Group

Organ Weights (g)—Female

Groups	Heart	Lung	Liver	Kidney	Spleen	Brain	Ovaries
VC	0.12 ± 0.02 (6)	0.37 ± 0.05 (6)	0.97 ± 0.06 (6)	0.4 ± 0.03 (6)	0.07 ± 0.001 (6)	0.45 ± 0.03 (6)	0.03 ± 0.01 (6)
TG	0.14 ± 0.012 (6)	0.32 ± 0.05 (6)	1.06 ± 0.04 (6)	0.38 ± 0.022 (6)	0.07 ± 0.002 (6)	0.45 ± 0.08 (6)	0.02 ± 0.01 (6)

Values are expressed in Mean ± S.D () No. of Animals
VC-Vehicle Control
TG-Theraupatic Group

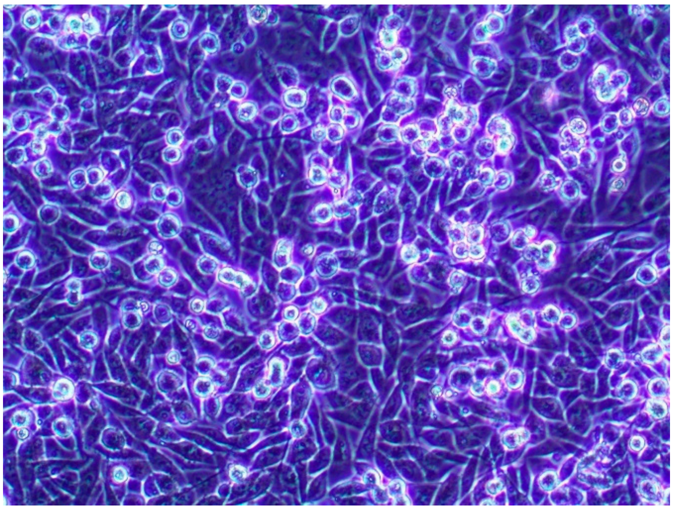

Figure S1. Morphology of PANC-1 cells.

Implantation of cells by S.C. (Subcutaneous) route

PANC-1 xenograft tumors were implanted using martigel in 6–8-week-old Female nude mice by implanting 1 × 10^6 PANC-1 cells s.c.

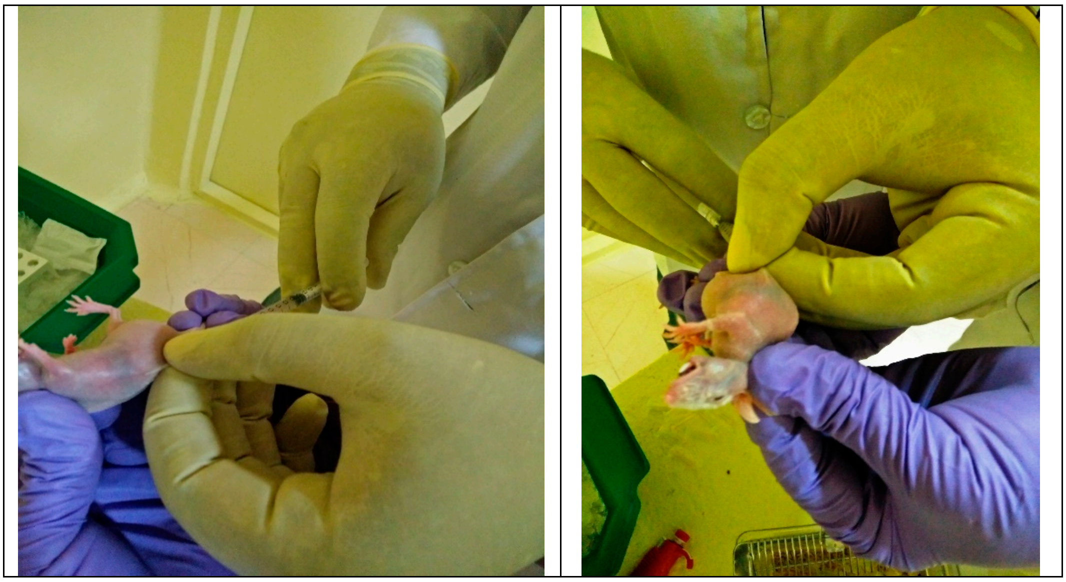

Figure S2. Implantation of cells through subcutaneous route (S.C.) using Matrigel.

PANC-1 cells Implantation for tumour development

We observed tumour development while implanting PANC-1 cell lines after 4 weeks of observation (by implanting 1 × 10[6] PANC-1 cells).

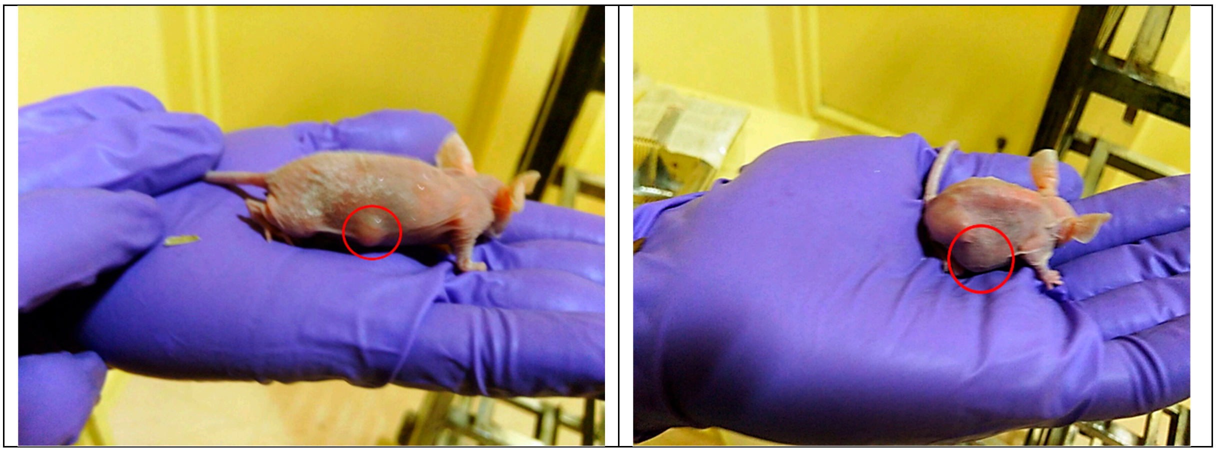

Figure S3. Tumor development of PANC-1 implanted cell lines in mice.

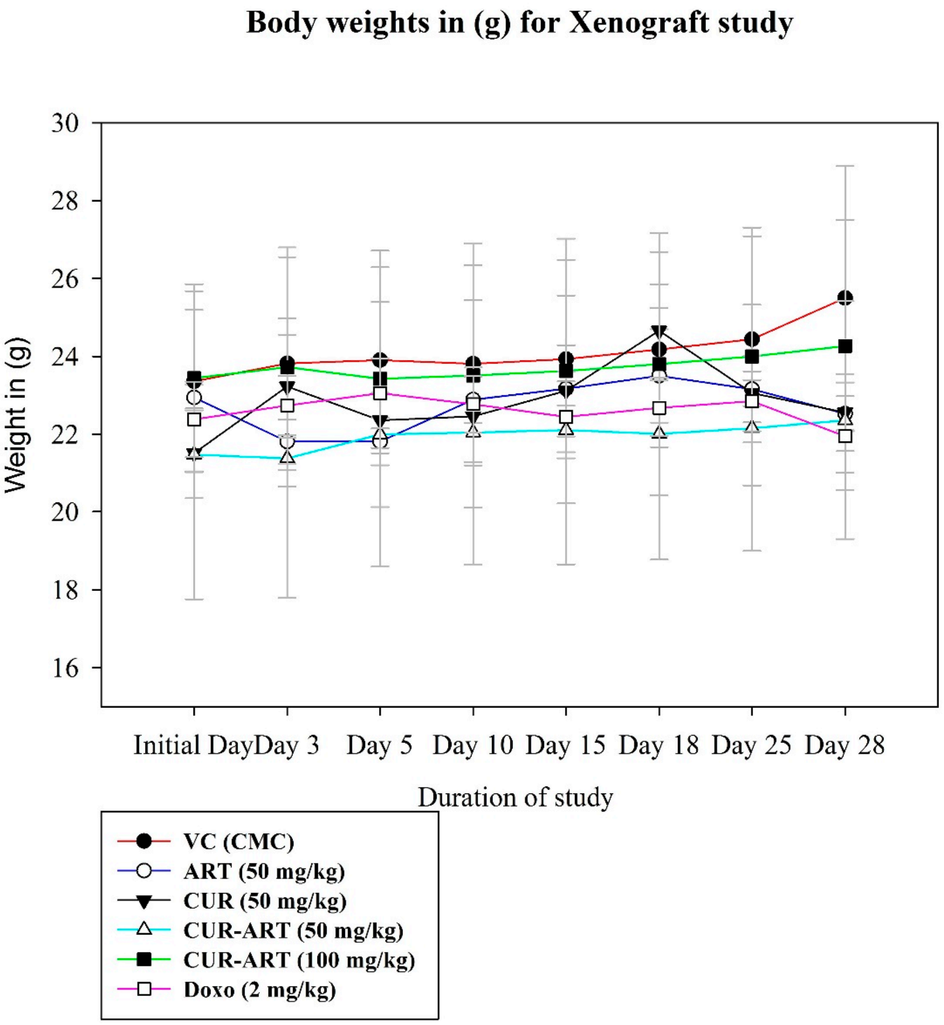

Figure S4. Body weight of selected nude mice for the xenograft studies.
